# Prevalence of Esophageal Eosinophilia, Eosinophilic Esophagitis, and Lymphocytic Gastritis in Children with Celiac Disease: A Saudi Tertiary Center Experience

**DOI:** 10.1155/2024/5541687

**Published:** 2024-01-09

**Authors:** Meshari A. Alaifan, Ammar Khayat, Rana Y. Bokhary, Abdulhameed Ibrahim, Yagoub Bin-Taleb, Bakr H. Alhussaini, Omar I. Saadah

**Affiliations:** ^1^Department of Pediatrics, Faculty of Medicine, King Abdulaziz University, Jeddah, Saudi Arabia; ^2^Pediatric Gastroenterology Unit, Department of Paediatrics, King Abdulaziz University Hospital, Jeddah, Saudi Arabia; ^3^Department of Pediatrics, College of Medicine, Umm Al-Qura University, Makkah, Saudi Arabia; ^4^Department of Pathology, Faculty of Medicine, King Abdulaziz University, Jeddah, Saudi Arabia; ^5^Department of Pediatrics, Hera Hospital, Makkah, Saudi Arabia

## Abstract

**Background:**

Celiac disease (CD) is an immune-mediated enteropathy that has been associated with other immune-related gastrointestinal disorders, such as eosinophilic esophagitis (EoE) and lymphocytic gastritis (LG). To our knowledge, this is the first study in Saudi Arabia that has described such an association.

**Aim:**

To evaluate the prevalence of EoE and LG in children and adolescents with CD.

**Methods:**

This was a retrospective cross-sectional study of all pediatric patients (aged 0–18 years) with CD following up at King Abdulaziz University Hospital, between January, 2014, and December, 2021. The study examined clinical, demographic, endoscopic, and histopathological data.

**Results:**

Seventy-five patients with CD were included in the analysis. The median age was 12 years (range, 2–18 years). Male constituted 54.7% of the overall cohort (*n* = 41). The most common clinical symptoms were short stature (54.7%), weight loss (34.7%), abdominal pain (33.3%), abdominal distension (29.3%), anorexia (29.3%), diarrhea (24%), and vomiting (21.3%). The esophageal biopsy results reported were basal cell hyperplasia in 24 patients (32.9%), esophageal eosinophilia in 23 patients (31.5%), and EoE in 3 patients (4.1%). The gastric biopsy results were normal in 40 patients (53.3%). The most common abnormality was chronic inactive gastritis with no *Helicobacter pylori* (HP) infection (16%). LG was found in 3 patients (4%).

**Conclusions:**

The prevalence of EoE in this cohort of patients with CD was lower than the prevalence recorded in a number of other studies. Further studies are needed to determine the effects of a gluten-free diet (GFD) on EOE and LG.

## 1. Introduction

Celiac disease (CD) is an immune-related enteropathy that can occur in genetically predisposed individuals following exposure to gluten and other environmental factors [1]. In a recent meta-analysis, the prevalence worldwide has been estimated to be around 0.7%, based on biopsy findings, and 1.4% based on serological testing [2]. In Saudi Arabia, a prevalence of biopsy-proven CD has been reported to be 1.5–2.2% in school-aged children [3, 4]. CD may present with intestinal manifestations, such as chronic diarrhea, abdominal pain, abdominal distension, vomiting, and failure to thrive, or extraintestinal manifestations, including osteopenia, iron deficiency anemia, rickets, short stature, and dental enamel defects [5, 6]. The only available treatment strategy is the implementation of a GFD.

CD was found to have an association with eosinophilic esophagitis (EoE) [7–12] and lymphocytic gastritis (LG) [13–16]. EoE is a chronic, immune-mediated esophageal disease, characterized clinically by symptoms related to esophageal dysfunction, and determined histologically by eosinophil-predominant inflammation, provided there is no evidence of systemic eosinophilia [17]. It has an estimated prevalence of 0.5–1 case per 1000 [18]. There are scant reports published locally regarding EoE, and there are no available data regarding prevalence in Saudi Arabia [19, 20]. The symptoms range from vomiting, food refusal, and failure to thrive in infants and to solid food dysphagia and food impaction in older children [20]. Treatment usually involves topical steroids, proton pump inhibitors, and food elimination diets [21].

Lymphocytic gastritis is characterized histologically by the accumulation of small lymphocytes in the surface and foveolar gastric epithelium [22]. LG is a rare disease with an estimated prevalence of 0.8–1.6% in adult patients with chronic gastritis [23]. It is commonly associated with HP infections [24, 25] and CD [7–13, 26].

The aim of this study was to determine the prevalence of EoE and LG in children and adolescents with CD.

## 2. Methods

### 2.1. Study Population

This was a retrospective cross-sectional study of all patients with clinical diagnosis of CD that was followed up at King Abdulaziz University Hospital between January, 2014, and December, 2021. We include all patients ≤18 years and patients that had an esophageal or gastric mucosal biopsy at the time of initial upper diagnostic endoscopy. Clinical, demographic, endoscopic, and laboratory data were extracted from the hospital health information system and patient medical files.

### 2.2. Histopathological Evaluation

Mucosal biopsy was obtained through upper gastrointestinal endoscopy performed under conscious sedation at the endoscopy unit. Biopsy specimens were taken from the esophagus (lower, middle, and upper), gastric body and antrum, and second part of the duodenum. The biopsy specimens were reviewed by a certified experienced gastrointestinal pathologist.

The diagnosis of CD was established according to the revised European Society of Pediatric Gastroenterology Hepatology and Nutrition (ESPGHAN) criteria [27], with demonstration of villous atrophy that was graded according to Marsh-Oberhuber classification [28], combined with positive celiac serology using antitissue transglutaminase (anti-tTG) antibody immune assay titres, which were positive (more than 20 units/ml) for all patients.

The diagnosis of esophageal eosinophilia was considered when eosinophils present in the mucosa of any number, while eosinophilic esophagitis (EoE) diagnosis was based on demonstration of eosinophils in the esophageal biopsy greater than 15 per high power field or presence of eosinophilic micr-abscesses [17].

The histopathological diagnosis of lymphocytic gastritis (LG) was established if 25 intraepithelial lymphocytes per 100 gastric epithelial cells infiltrated the surface epithelium [24], in the absence of HP organisms.

### 2.3. Statistical Analysis

Descriptive statistics were used to present patient data. Categorical data were expressed as score and percentage, and continuous data were expressed as mean and standard deviation (SD).

### 2.4. Ethical Considerations

The study has been approved by the Research Committee of the Biomedical Ethics Unit, at King Abdulaziz University (Reference no. 571−21). The ethical considerations were followed in accordance with the Declaration of Helsinki.

## 3. Results

### 3.1. Clinical and Demographic Data

A total of 75 patients with biopsy-proven diagnosis of CD were included in the analysis. The median age was 12 years (range, 2–18 years). Male constituted 54.7% of the total cohort (*n* = 41). The most common clinical symptoms were short stature (54.7%), weight loss (34.7%), abdominal pain (33.3%), abdominal distension (29.3%), loss of appetite (29.3%), diarrhea (24%), and vomiting (21.3%). Most patients (48%) were categorized as Marsh 3b enteropathy score.  Table 1 summarizes baseline clinical, demographic, and laboratory findings at diagnosis.

## 4. Outcomes

### 4.1. Esophageal Involvement

**Figure 1:**
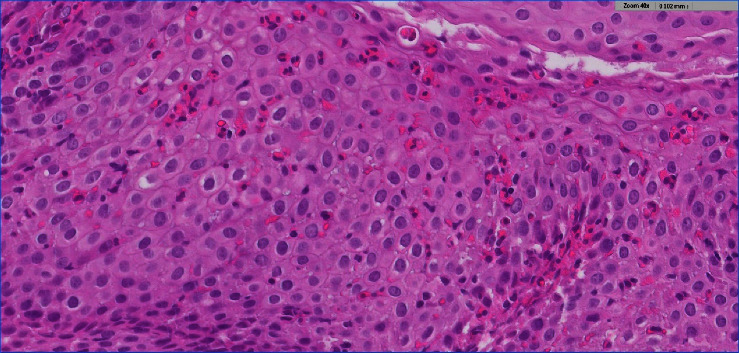
Histological features of eosinophils esophagitis. A close view demonstrating the high number of intraepithelial eosinophils seen in some cases. Eosinophils create small collections near the surface “eosinophils microabscesses” and are degranulating (hematoxylin and eosin stain, 40x).

Esophageal biopsy was performed on 73 patients (97.3%). Of the total patients that underwent esophageal biopsy, basal cell hyperplasia was found in 24 patients (32.9%), esophageal eosinophilia were reported in 23 patients (31.5%), and 3 patients (4.1%) had eosinophil counts exceeding 15 per high power field, compatible with the definition of eosinophilic esophagitis. The histological features of EoE are shown in Figure 1.

### 4.2. Gastric Involvement

**Figure 2:**
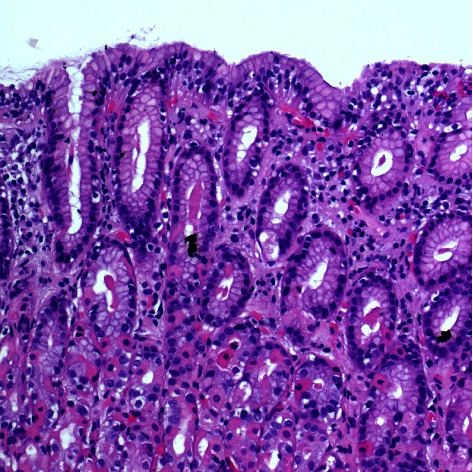
Lymphocytic gastritis. Increased number of intraepithelial lymphocytes can be appreciated more in the surface epithelium (hematoxylin and eosin stain, 10x).

Gastric biopsy was undertaken in 75 patients. The biopsy results were reported as normal in 40 patients (53.3%). The most common abnormality was chronic inactive gastritis with no HP infection (16%). Lymphocytic gastritis was found in 3 patients (4%), shown in Table 2. The histological features of lymphocytic gastritis are shown in Figure 2.

### 4.3. Endoscopic Findings

The most common findings of the duodenal mucosal findings during endoscopy were the presence of fissures or grooves between mucosal folds in 36% (*n* = 27) and scalloping of the Kerckring folds in 29.3% (*n* = 22). Gastric mucosa was normal in 76% (*n* = 57), followed by mucosal erythema in 16% (*n* = 12). The esophageal mucosa looked normal in 85% (*n* = 62), furrowing in 8.2% (*n* = 6), and erythematous in 6.8% (*n* = 5).  Table 3.

## 5. Discussion

The pathophysiology of CD is complex but is known to involve activation of intestinal T cells, following exposure to gluten [29, 30]. This is reflected histologically by the characteristic lymphocytic infiltration of the surface epithelium, all of which are of T cell (CD3) subtypes, based on immunohistochemical stains [31, 32]. The pathophysiology of LG is not fully understood but its association with CD or HP infection may suggest an overlapping role of T cells in these conditions [33]. Similar to CD, it is characterized histologically by lymphocytic infiltration of the surface as well as crypt epithelium [14]. The lymphocytes are all of T cell (CD3) subsets in immunohistochemical studies, which again hints at a possible common pathophysiologic pathway with CD [33]. A number of other gastrointestinal lymphocytic disorders have been reported with increasing frequency in association with CD, such as lymphocytic colitis, which yet again may share a common pathophysiology [34, 35]. Mucosal T cells in general have an important role in maintaining homeostasis of the adaptive immunity of the gastrointestinal tract, preventing microbial invasion while avoiding unnecessary immune activation against different food antigens [36]. Disruption of this balance results in various autoimmune disorders, such as inflammatory bowel diseases [36].

EoE is also an immune-mediated food antigen-triggered disorder, which has a well-established association with asthma, atopic conditions, and eosinophilic gastroenteritis [37]. The association between EoE and CD although often described is not well understood [7–12]. Eosinophils have a well-known role in allergic conditions, as well as parasitic infections [38, 39]. In recent years, research has shed some light on a wider range of functions of eosinophils in both the innate as well as the adaptive immune system, including their role as antigen presenting cells for T cells [38, 39]. Lingblom et al. have demonstrated that eosinophils have the ability to suppress T cell activity in the esophagi of normal individuals, but this function is impaired in patients with EoE [40]. Whether this interaction between eosinophils and T cells reflects the association between EoE and other “T cell disorders,” such as CD or lymphocytic gastritis, is unclear [7–12, 40].

The overlapping pathophysiology between CD on the one hand and EoE or LG on the other may be reflected in the prevalence of the latter disorders in the context of CD, where the odds ratios are increased in the setting of CD compared to the general population [9, 16]. The prevalence of EoE and LG in patients with CDin the present cohort was lower than the prevalence reported in a number of other studies [13–16]. This may be due to differences in CD severity among the various cohorts and age groups [7–12]. In a study by Ahmed et al. [7] involving a large cohort, it was shown that the odds ratio of EoE is increased for adults but for not children with CD, which was again demonstrated in the study by Ahmed et al. [7, 9].

The higher odds ratio for EoE or LG in the context of CD suggests the importance of clinical debate on obtaining a full set of esophageal and gastric biopsies in patients undergoing initial screening endoscopy for CD [27]. It has been found that the correlation between individual endoscopic findings for EoE and histopathology is poor, owing to low sensitivities (40–50%) and high false-negative rates (5–32%) [41], and this rate is even higher in the present cohort. Similarly, endoscopic findings for chronic gastritis and LG have low sensitivities (18–63%), and thus often have a poor correlation with histopathology [42, 43]. This high false-negative rate supports obtaining esophageal and stomach biopsies routinely in patients with CD, even when the mucosal lining looks normal endoscopically, although this is still controversial among many clinicians and scientists [7].

The strengths of the current study include the availability of histopathologic diagnosis and a relatively good sample size. The study may be limited by an absence of follow-up data to assess treatment response in order to aid diagnosis. Given that EoE and LG are histologically diagnosed, further follow-up requires endoscopy. It poses a challenge, given the procedural and anesthetic risks, especially in the pediatric age group.

## 6. Conclusion

The prevalence of both EoE and LG in the present cohort of Saudi Arabian children with CD seems to be lower than the prevalence reported in other similar studies. Further longitudinal studies are needed to determine the effects of GFD on EoE and LG.

## Figures and Tables

**Table 1:** Baseline clinical and demographic characteristics of the study cohort at diagnosis (*n* = 75).

Variable	Mean ± SD, *n* (%)
*Demographics*	
Age (years)	12.1 ± 4.5
Male, gender	41 (54.7%)
*Clinical manifestations*	
Short stature	41 (54.7%)
Weight loss	26 (34.7%)
Abdominal pain	25 (33.3%)
Abdominal distension	22 (29.3%)
Loss of appetite	22 (29.3%)
Diarrhea	18 (24%)
Vomiting	16 (21.3%)
Pallor	10 (13.3%)
Joint and bone pain	9 (12%)
Mouth ulcers	8 (10.7%)
Arthritis	6 (8%)
Lethargy	6 (8%)
*Laboratory findings*	
Tissue transglutaminase-IgA	171 ± 90.5
Hemoglobin (g/dL)	12 ± 1.6
Albumin (g/L)	37.7 ± 6.8
25-Hydorxyvitamin (ng/mL)	47.6 ± 28
*Histopathological marsh scoring*	
Marsh 3a	15 (20%)
Marsh 3b	36 (48%)
Marsh 3c	24 (32%)

**Table 2:** Gastric biopsy histopathological findings of 75 patients with CD.

	Number (%)
Normal	40 (53.3%)
Chronic inactive gastritis without HP	12 (16%)
Chronic inactive gastritis with HP	2 (2.7%)
Chronic active gastritis without HP	8 (10.7%)
Chronic active gastritis with HP	10 (13.3%)
Lymphocytic gastritis	3 (4%)

**Table 3:** Endoscopic findings of the esophagus, stomach, and duodenum of the study cohorts.

Findings	*N* (%)
*Esophageal findings (n* *=* *73)*	
Normal	62 (85%)
Erythema	5 (6.8%)
Furrowing	6 (8.2%)
*Gastric findings (n* *=* *75)*	
Normal	57 (76%)
Erythema	12 (16%)
Antral nodularity	4 (5.3%)
Erosions	2 (2.7%)
*Duodenal findings (n* *=* *75)*	
Normal	10 (13.3%)
Erythema	9 (12%)
Mosaic or micronodular appearance	3 (4%)
Fissuring or grooving between folds	27 (36%)
Loss or reduction of folds	4 (5.4%)
Scalloping of kerckring folds	22 (29.3%)

## Data Availability

Data are stored in a secured flash drive in the University campus. The data used to support the findings of this study can be made available upon request.
